# In Vitro Assessment of Tobacco Smoke Toxicity at the BBB: Do Antioxidant Supplements Have a Protective Role?

**DOI:** 10.1186/1471-2202-12-92

**Published:** 2011-09-24

**Authors:** Mohammed Hossain, Peter Mazzone, William Tierney, Luca Cucullo

**Affiliations:** 1 Cerebrovascular Research Department of Pulmonary Medicine, Cleveland Clinic, Cleveland, OH 44195 USA; 2 Department of Cell Biology Department of Pulmonary Medicine, Cleveland Clinic, Cleveland, OH 44195 USA; 3 Department of Pulmonary Medicine, Cleveland Clinic, Cleveland, OH 44195 USA; 4 Case Western Reserve University, Cleveland, OH 44106 USA; 5 Pharmaceutical Sciences, Texas Tech University Health Sciences Center, Amarillo, TX 79106 USA

## Abstract

**Background:**

Tobacco smoke (TS) contains highly reactive oxygen species (such as hydrogen peroxide, peroxynitrite, etc), which cause oxidative damage in vascular tissue and may exacerbate inflammatory events leading to the blood-brain barrier damage (BBBD) which accompanies the development of a variety of neurological disorders. Smokers often have elevated leukocyte counts (primarily neutrophils and monocytes), and significant decreases in plasma alpha-tocopherol (vitamin E) and ascorbic acid (vitamin C) levels due to increased anti-oxidative mobilization in response to oxidative stress evoked by TS. For this purpose, using static culture systems and a well-established dynamic *in vitro *BBB model (DIV-BBB) we tested the hypothesis that antioxidant vitamin supplementation (E and/or C) can protect the BBB during exposure to whole soluble TS.

**Results:**

TS exacerbates inflammatory events and leads to endothelial overexpression of vascular adhesion molecules (VCAM-1, P-selectin and E-selectin), release of pro-inflammatory cytokines (TNF-α and IL-6) and nitric oxide (NO), release and activation of matrix metalloproteinases (MMP-2 and MMP-9), monocytic maturation into macrophages, and adhesion to the vascular endothelium. Furthermore, TS altered the normal glucose metabolic behaviour of *in vitro *BBB capillaries and caused a period of transient anaerobic respiration to meet the cellular bioenergetic demand. Pre-treatment with antioxidant vitamins (C and/or E) effectively reduced the pro-inflammatory activity associated with TS, protecting the viability and functions of the BBB.

**Conclusion:**

Our results have shown that loss of endothelial viability as well as BBB function and integrity caused by TS exposure can be prevented or at least reduced by normal physiologic concentrations of antioxidant vitamins *in vitro*.

## Background

### Tobacco Smoke affects vascular endothelial physiology

Active and passive tobacco smoke are associated with dysfunction of vascular endothelial physiology [1-13] in a causative and dose-dependent way. In the brain, TS increases the risk of silent cerebral infarction (SCI) [14] and stroke which by approximately 50% [15,16] due to its; pro-coagulant and atherogenic effects [17,18]. The risk increases proportionally with the number of cigarettes smoked [19]. Furthermore, oxidative stress caused by the exposure to TS can facilitate the pathogenesis and progression of several neurological disorders including Parkinson's and Alzheimer's diseases [20-24]. Previous study has shown that chronic smokers have a higher incidence of small vessel ischemic disease (SVID) than non-smokers [25]. SVID is a pathological condition characterized by leaky brain microvessels and loss of BBB integrity. Since TS generates superoxide and other ROS, some of the adverse effects of smoking may result from oxidative damage to ECs, [4,12,13,26-29], which may be aggravated by nitric oxide (NO) and antioxidant depletion (e.g., ascorbic acid). Recent observations strongly suggest that ROS are key mediators of BBB breakdown [25,26,30-33].

While the harmful effects of smoking on public health have been well demonstrated and detailed in many organs, the BBB has received much less attention despite the strong evidence for an association between tobacco smoke and vascular impairment [28,34]. Our recently published data [25] have shown that TS promotes the pro-inflammatory activation of BBB EC and the release of pro-inflammatory mediators. This occurred in the absence of other pro-inflammatory stimuli and independently from the presence of peripheral blood cells. Furthermore; TS can significantly exacerbate the loss of BBB integrity caused by concurrent cerebrovascular rheological alterations, [25,35] facilitating the further pathogenesis and progression of secondary brain injuries.

### Tobacco smoke: A harmful cloud of Free Radicals

The role of free radicals in the pathogenesis of smoking-related diseases has been substantiated by a large number of studies. A free radical is a highly unstable and reactive molecule containing one or more unpaired electrons. Free radicals, despite being essential for biological systems [36] have a potential to cause extensive oxidative damage to cells and tissues if their levels become excessive [21,22,27,37-41]. At the vascular level free radicals can lead to oxidative damage of endothelial cells [12,13] (e.g., DNA strand breakage [3,42-44]) and inflammation [3]. Free radicals arise normally during cell metabolic activity and are also purposefully produced by immune cells to neutralize potentially harmful pathogens such as bacteria and viruses. *In vivo*, under normal circumstances the oxidative stress caused by naturally generated free radicals is counterbalanced by antioxidants [45,46] (e.g., alpha-tocopherol, ascorbic acid, beta-carotene, glutathione, catalase, superoxide dismutase, etc.). However, environmental factors including active and passive tobacco smoking (a major exogenous source of free radicals contained in both gas phases and tar [47-49]) can spawn these highly reactive oxygen species (ROS; e.g., hydrogen peroxide, epoxides, nitric oxide (NO), nitrogen dioxide, peroxynitrite (ONOO-), etc [49]) beyond the levels which the human body can eliminate effectively. In fact, several studies have shown that 1) chronic smokers suffer of antioxidants shortage caused by the increased demand that the ROS derived from TS place on their cells [13,50,51]; 2) antioxidant supplementation reduces the oxidation and inflammation induced by tobacco smoke in animals and cells [13,50,52-55]. In response to these studies, the Food and Nutrition Board of the National Academy of Sciences has established a higher Recommended Dietary Allowance (RDA) of vitamin C for smokers, (more than 200 mg/day) versus the recommended 90 mg/day for non-smokers.

### Is there a role for Antioxidant Vitamins Supplementation?

Antioxidants such as vitamin C (ascorbic acid) and E (α-tocopherol) are compounds that hinder the oxidative processes and thereby delay or prevent oxidative stress. Although there is still no unequivocal evidence that an increased intake of antioxidant nutrients can fully counteract TS toxicity, there are many supporting data suggesting that antioxidants may prove to be effective scavengers of exogenous-derived ROS [56]. Previously, vitamin C has been shown to act not only as a potent reducing agent but also to play a role (currently not yet clearly understood) in modulating the production of lymphocytes and cytokines, the activity of phagocytes, and the expression of a number of cell adhesion molecules in monocytes [57]. Furthermore, vitamin C seems to prevents histamine release and increases the detoxification of histamine [58], thus acting as an anti-inflammatory agent as well as a potent antioxidant. Considering the major role played by ROS in the pathogenesis of BBB impairment as well as that of neurological disorders [21-23,32,59] we evaluated the independent and complementary effectiveness of antioxidants vitamins supplementation, such as vitamin C (protection against protein oxidation [60-62] and vitamin E (counteract lipid peroxidation [63-65]), in shielding the BBB from the chronic exposure to TS-generated oxidative stress.

## Results

### Tobacco Smoke pro-inflammatory response induced in brain microvascular endothelial cells was reduced by antioxidant vitamins supplementation

**Figure 1:**
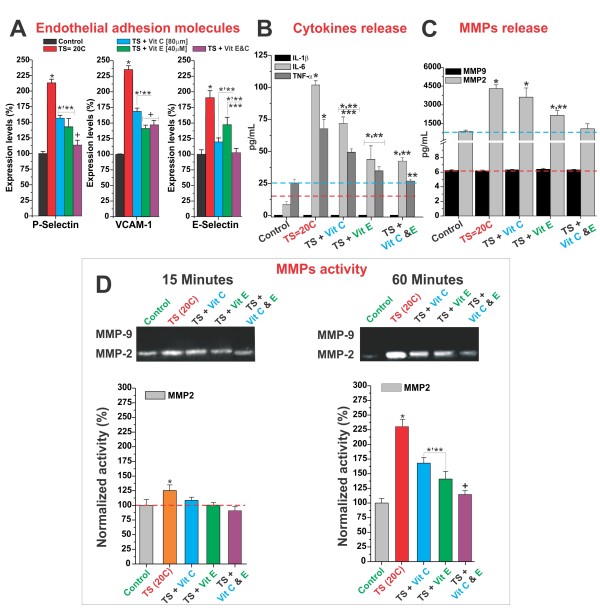
**Antioxidant vitamins administration reduced TS-dependent the pro-inflammatory activation of BBB endothelial cells**. Chronic exposure of confluent cultures of HBMEC to TS levels equivalent to 20 cigarettes/day increased the number of HBMEC cells expressing of endothelial adhesion molecules E-selectine, P-selectine and VCAM-1. However, the pro-inflammatory effect of TS was partially counteracted by pre-treatment with the antioxidant vitamin C and E (Panel **A**), which as expected reduced the overall release of pro-inflammatory cytokines IL-6 and TNF-α (Panel **B**). MMP-2 release and activity were also significantly augmented by TS exposure (Panel **C**). MMP-2 release and activation following TS exposure were most effectively prevented by vitamin C and E co-administration. All the described experiments were performed in quadruplicates (n = 4). The asterisk "*" indicates a statistically significant difference (p < 0.05) versus control. Multiple asterisks indicate a statistically significant difference (p < 0.05) versus other treatments. The plus sign "+" indicates a statistically significant difference (p < 0.05) versus the control as well as all the other treatments.

Chronic exposure of confluent cultures of human brain microvascular endothelial cells (HBMEC) to TS (equivalent to 20 cigarettes/day; see in Material and Methods for details) resulted in a significant increase in the number of HBMEC cells expressing the pro-inflammatory adhesion molecules P-selectin, VCAM-1 and E-selectin the expression levels of P-selectin, VCAM-1 and E-selectin (112.5% ± SEM 8.2, 144.5% ± SEM 6.3, and 84.3% ± SEM 12.4 respectively) as compared to sham-smoke-exposed controls (Figure 1A). The augmented expression of these pro-inflammatory adhesion molecules was paralleled by over a 10 fold increase (from 9.1 ± SEM 2.1 to 103.3 ± SEM 4.3 pg/mL) of interleukin-6 (IL-6) levels in the medium, and over a 2 fold increase in that of **tumor necrosis factor**-**alpha **(TNF-α) (from 25.8 ± SEM 4.5 to 67.2 ± SEM 9.0 pg/mL). IL-1β levels were unaffected (Figure 1B). Exposure to TS also increased the release of matrix metalloproteinases-2 (MMP-2) from 775 ± SEM 98 to 4344 ± SEM 235 pg/mL. No release of MMP-9 was detected (Figure 1C). Gelatin zymography of electrophoresed medium samples showed a small (28.3 ± SEM 9.0%) yet significant increase in MMP-2 activity 15 minutes following TS exposure, which substantially increased to 132.7 ± SEM 14.3% at 60 minutes (Figure 1D).

Pre-treatment with vitamins C and/or E (final physiological concentrations ≅ 80 and 40 μM respectively) significantly reduced the pro-inflammatory activity of TS on HBMEC. Specifically the over-expression of endothelial adhesion molecules during TS exposure (Figure 1A) was significantly reduced. This was paralleled by a decrease in the release of IL-6 and TNF-α (Figure 1B). MMP-2 release (Figure 1C) and activity (Figure 1D) were reduced in cultures pre-treated with vitamin E and E+C (combined treatment) but not vitamin C alone. Overall, the combined treatment showed the most effective protection against TS exposure.

### Monocytes activation by Tobacco Smoke was partially inhibited by pre-treatment with vitamin C and/or vitamin E

**Figure 2:**
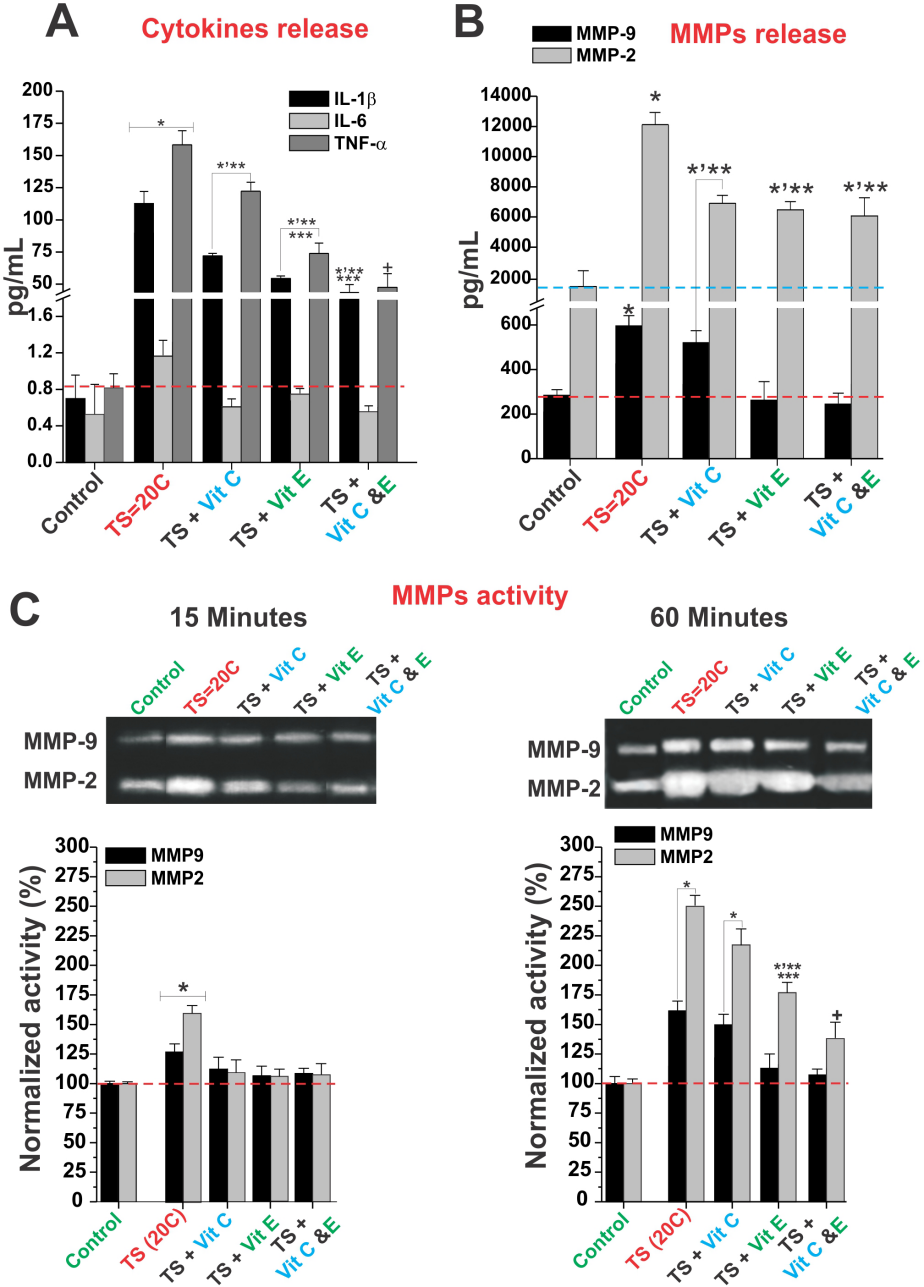
**Administration of vitamins E and C reduced the immune activation of monocytes and the subsequent release of pro-inflammatory mediators during TS exposure**. Chronic TS induced a marked increase in the medium content of IL-1b and TNF-α (Panel **A**), which were partially offset by the administration of antioxidant vitamins E and C. The medium content (Panel **B**) and activity (Panel **C**) of MMP-2 and-9 was also substantially increased by TS exposure. However, this was partially counteracted by vitamin E pre-treatment (alone) and vitamin E in combination with vitamin C. Vitamin C alone was only marginally effective. All the described experiments were performed in quadruplicates (n = 4). The asterisk "*" indicates a statistically significant difference (p < 0.05) versus control. Multiple asterisks indicate a statistically significant difference (p < 0.05) versus other treatments. The plus sign "**+**" indicates a statistically significant difference (p < 0.05) versus the control as well as all the other treatments.

Our results showed a very marked increase of TNF-α (from 0.81 ± SEM 0.14 to 163.11 ± SEM 7.83 pg/mL) and IL-1b (from 0.66 ± SEM 0.21 to 113.59 ± SEM 8.15 pg/mL) levels in the culture medium of THP-1 monocytes upon exposure to TS (see in Materials and Methods for details) (Figure 2A). IL-6 level was only moderately effected but the difference was not statistically significant. In parallel, we observed a significant increase in the medium levels (Figure 2B) of MMP-9 and MMP-2 (from 285.71 ± SEM 22.44 to 596.55 ± SEM 49.68 pg/ml and from 1638.56 ± SEM 1095.34 to 12089.50 ± SEM 912.28 pg/mL respectively). Increased MMPs activity (MMP-2 and MMP-9) was already evident after 15 minutes of TS exposure (62.9 ± SEM 4.4% and 26.4 ± SEM 3.8% respectively; see Figure 2C) and became significantly more prominent at 60 minutes (151.9 ± SEM 8.8% and 60.5 ± SEM 5.6% respectively).

TS-triggered release of pro-inflammatory cytokines was most significantly reduced by pre-treatment with vitamins C and E. In contrast to vitamin C, vitamin E alone also demonstrated significant anti-inflammatory protection.

The TS-induced release of MMP-2 and MMP-9 was also decrease by the antioxidant vitamins however, there were no statistical significant differences in terms of efficacy between individual (Vit. C or E) and combined pre-treatments. By contrast, the most effective blockade of MMPs activation was attained using combined (C + E) vitamins supplementation (Figure 2C). Vitamin E alone was also effective while only short term MMPs activity reduction was observed when using vitamin C (Figure 2C left panel).

### Antioxidant vitamins reduced endothelial-monocytes adhesion and synergistically decreased the release of pro-inflammatory mediators during TS exposure

**Figure 3:**
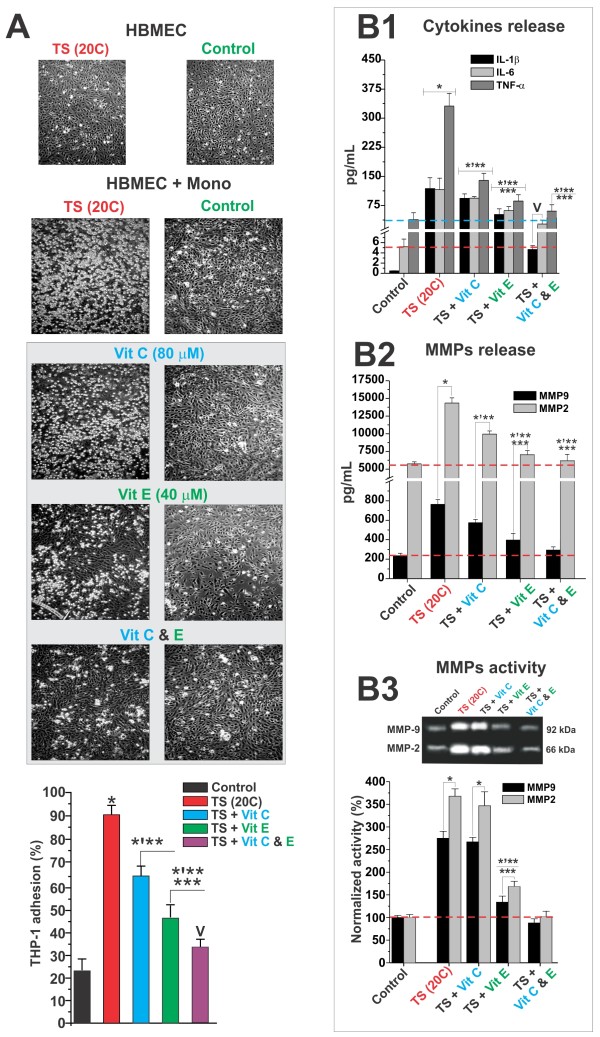
**Antioxidant vitamins reduced the synergistic pro-inflammatory activation on HBMEC in presence of THP-1 cells during TS exposure**. Visual inspection and quantification of adherent monocytes showed a dramatic increase in the number of monocyte-endothelial bonded cells following TS exposure (Panel **A**). Leukocyte-endothelial interactions during TS exposure were prevented by pre-treatment with physiological concentrations of vitamins E and (to a lesser extent). Analysis of the medium content showed that pre-treatment with antioxidant vitamins tested herein also reduced the release of IL-1β, IL-6 and TNF-α (Panel **B1**), that of MMP-2 and MMP-9 (Panel **B2**) and also reduced the MMP-2 and -9 activation (Panel **B3**). Note that the anti-inflammatory protection provided by pre-treatment with vitamin E alone and that of vitamin E and C combined, was significantly more effective (by magnitude and duration) than that of vitamin C alone. All the described experiments were performed in quadruplicates (n = 4). The asterisk "*" indicates a statistically significant difference (p < 0.05) versus control. Multiple asterisks indicate a statistically significant difference (p < 0.05) versus other treatments. The plus sign "V" indicates a statistically significant difference (p < 0.05) versus the control as well as all the other treatments.

HBMEC and THP-1 cells cultures were individually exposed to TS as described above. Parallel sham-smoke cultures were used as control. After the TS exposure cycle was completed, the wells containing the endothelial cells underwent a wash out and medium replacement using a TS-free one. In parallel, THP-1 cells were isolated by centrifugation, then re-suspended (2 × 10^5 ^cells/mL) with the endothelial cells and left to undergo adhesion for one hour without agitation. Non-adherent monocytes were then washed away by gentle shaking and the medium was collected for a content analysis of pro-inflammatory mediators (cytokines and MMPs). Visual inspection (Figure 3A upper panels) and quantification of adherent monocytes (Figure 3A lower panel) showed a dramatic increased in the percentage of monocytes bonded to endothelial cells following TS exposure (90.3 ± SEM 5.0%) in comparison to sham-smoke cultures (23.6 ± SEM 5.2%). Pre-treatment with normal physiological concentrations of vitamins E and (to a lesser extent) C reduced the percentage of adherent cells in TS pre-exposed cultures (66.2 ± SEM 4.6% and 47.4 ± SEM 6.7% respectively). The combined pre-treatment with both vitamin E and C was the most effective (36.8 ± SEM 4.0%). As previously observed in addition to enhanced endothelial-monocytes adhesion, TS exposure lead to a significant increase in the medium content of IL-1β, IL-6 and TNF-α (0.58 ± SEM 0.5 to 112.2 ± SEM 29.01; 5.01 ± SEM 2.2 to 109.3 ± SEM 33.7; 62.5 ± SEM 29.8 to 328.6 ± SEM 34.9 pg/mL respectively). Antioxidant vitamin pre-treatment was effective (at various levels) in reducing the TS-induced release of these pro-inflammatory cytokines (Figure 3B1). As expected, culture medium analysis showed that the interaction between TS-activated THP-1 cells with TS-activated HBMEC prompted a synergistic increase in the release and activation of MMP-2 and MMP-9 (Figure 3B2 and 3B3). Vitamin C reduced the release of MMP-2 and MMP-9 but was ineffective in blocking their corresponding activation as demonstrated by the zymogram gel analysis of medium content (Figure 3B3, upper panel). By contrast the anti-inflammatory protection provided by vitamin E and the combined treatment (C + E) were significantly more effective.

### Ascorbic acid and α-tocopherol protect the BBB viability from the harmful exposure to Tobacco Smoke

**Figure 4:**
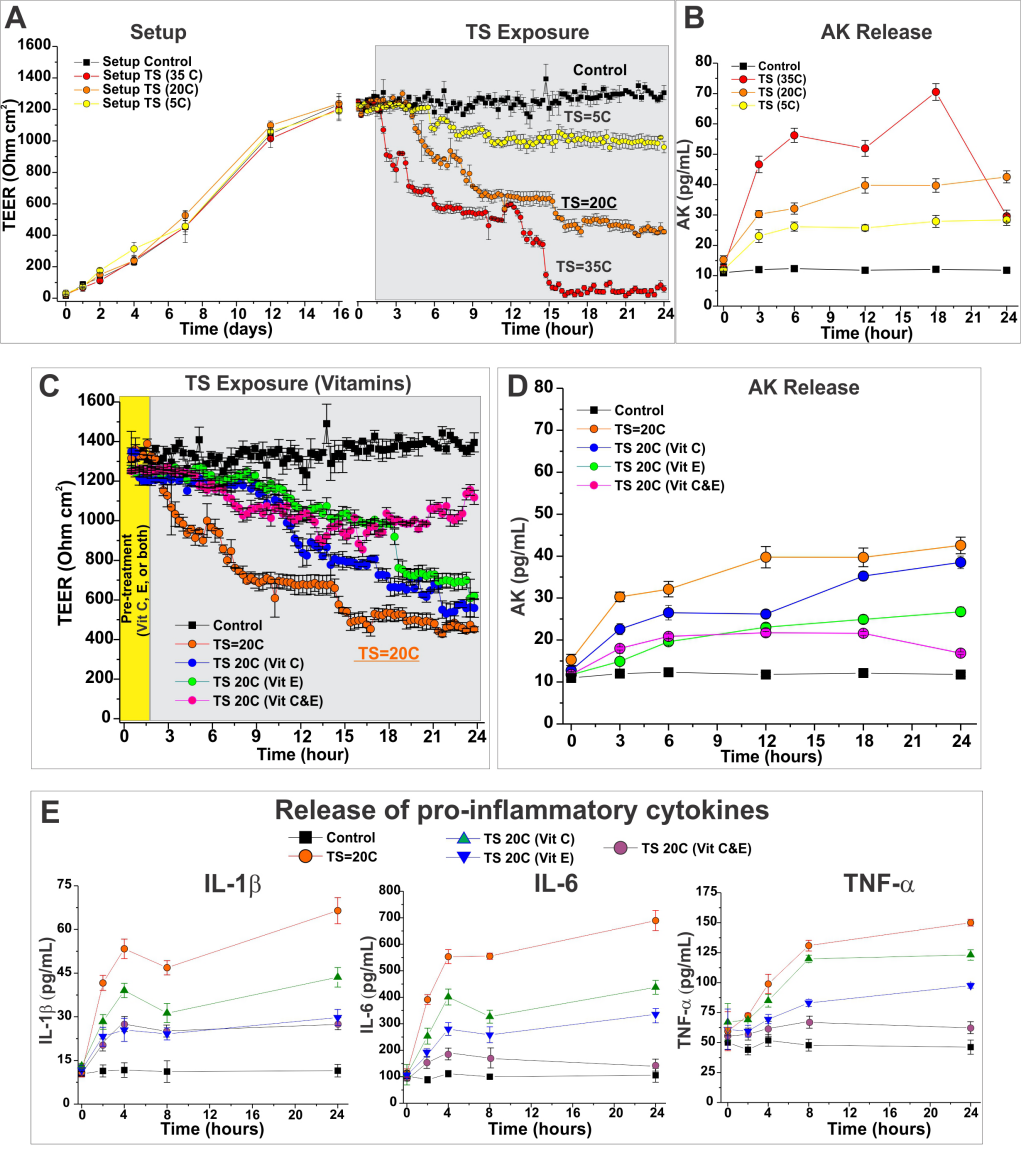
**Antioxidant vitamins protect against TS-dependent loss of BBB viability and integrity**. Under the exposure to pulsatile flow in presence of abluminal astrocytes and circulating THP-1 cells the vascular endothelium formed a stable highly impermeable barrier (TEER ≈ 1200 Ohm/cm^2^) (**Panel A **-left). Exposure to TS at concentrations equivalent to yield by 20 cigarettes/day or more higher caused a loss of BBB integrity (**Panel A **-right) which was paralleled by a significant and proportional (to TS exposure) release of AK (**Panel B**). The loss of BBB integrity during TS exposure (≅ 20 cigarettes) was most significantly reduced by the combined pre-treatment with vitamins C and E (**Panel C**) as also demonstrated by the parallel decrease in AK release (**Panel D**) and that of pro-inflammatory mediators IL-1β, IL-6 and TNF-α (**Panel E**). The described experiments were performed in quadruplicates (n = 4).

The effect of TS exposure (equivalent to that generated by 5, 20, and 35 cigarettes/day (5C, 20C, 35C) respectively) on BBB integrity was evaluated fully established DIV-BBB [66-68] modules (Figure 4A). Sham-TS-exposed modules were used as control. TS levels equivalent to 5C did not significantly impaired BBB integrity as demonstrated by TEER (Figure 4A left panel) and Adenylate kinase (AK) release measurements (Figure 4B). In contrast, exposure to higher TS levels (20 and 35C) resulted in rapid and dose-dependent loss of BBB integrity, which was accompanied by a significant increase of AK release when compared to controls, and to the release of AK that followed the exposure to a low level of TS (Figure 4A and 4B). We assessed the protective efficacy of antioxidant vitamins against the BBB exposure to TS levels equivalent to 20C/day. BBB integrity was partially protected against TS exposure-related damage by vitamin E and C as demonstrated by TEER (Figure 4C) and AK release (Figure 4D) measurements. The independent administration of these substances resulted in a delayed and a less severe loss of BBB viability. Note that the co-administrations of these vitamins provided the most significant protection. In addition to BBB deterioration during TS exposure, the analysis of the media samples showed a statistically significant increase in the levels of IL-1β, IL-6, and TNF-α (Figure 4E). Administration of antioxidant vitamins significantly reduced the TS-induced release of these pro-inflammatory cytokines with the combo approach being the most effective.

### Hydrogen peroxide impairs BBB integrity: Protective role of antioxidant vitamins

**Figure 5:**
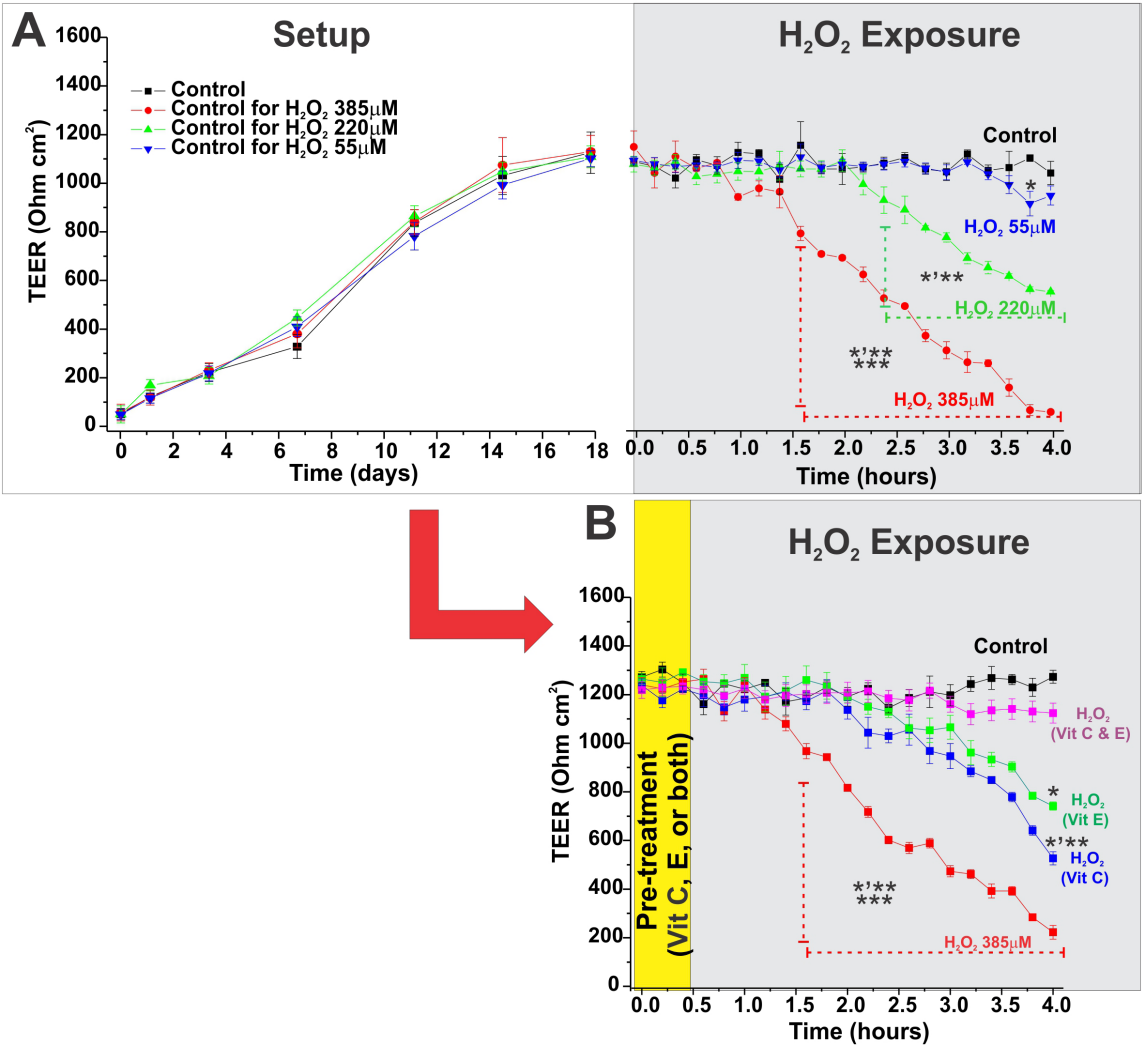
**Exposure to hydrogen peroxide affects BBB viability and integrity**. **(Panel A) **Under the exposure to pulsatile flow in presence of abluminal astrocytes the vascular endothelium formed a stable highly impermeable barrier (TEER ≈ 1200 Ohm/cm^2^) (**Panel A **left side). Exposure to H_2_O_2 _equivalent to that contained in 10 and 20 cigarettes determined a loss of BBB integrity (**Panel A **right side). Loss of BBB integrity and viability at the highest H_2_O_2 _level of exposure was partially prevented to a different extent by pre-treatment with antioxidant vitamins (C, E, and combined treatment, **Panel B**). The described experiments were performed in quadruplicates (n = 4). The asterisk "*" indicates a statistically significant difference (p < 0.05) versus control. Multiple asterisks indicate a statistically significant difference (p < 0.05) versus other treatments.

The effect of hydrogen peroxide (H_2_O_2_, one of the many redox active substances contained in cigarette smoke; 2-4 μ M H_2_O_2_/mg of tar [69,70]) exposure on BBB integrity was assessed in the DIV-BBB. Three levels of H_2_O_2 _exposure comparable to that yield by 5, 20, and 35 2R24 research cigarettes (55, 220, and 385 μM respectively) were tested. Sham- H_2_O_2_-exposed modules were used as control. Exposure to H_2_O_2 _equivalent to that generated by 5 cigarettes (55 μM) did not affect BBB integrity (Figure 5A-**right panel**) as demonstrated by TEER measurements. In contrast, exposure to higher levels of H_2_O_2 _(110 and 220 μM respectively) resulted in rapid and dose-dependent loss of BBB integrity. Pre-treatment with vitamin E and C provided only a partial protection against oxidative damage to the BBB (Figure 5B). Note that the efficacy of the antioxidative protection was assessed against the effect of the highest of the 3 levels of H_2_O_2 _exposure (220 μ M). Co-administration of vitamins C and E fully prevented the loss of BBB integrity and viability.

### Tobacco smoke can impair BBB viability through alteration in nitric oxide bioavailability

**Figure 6:**
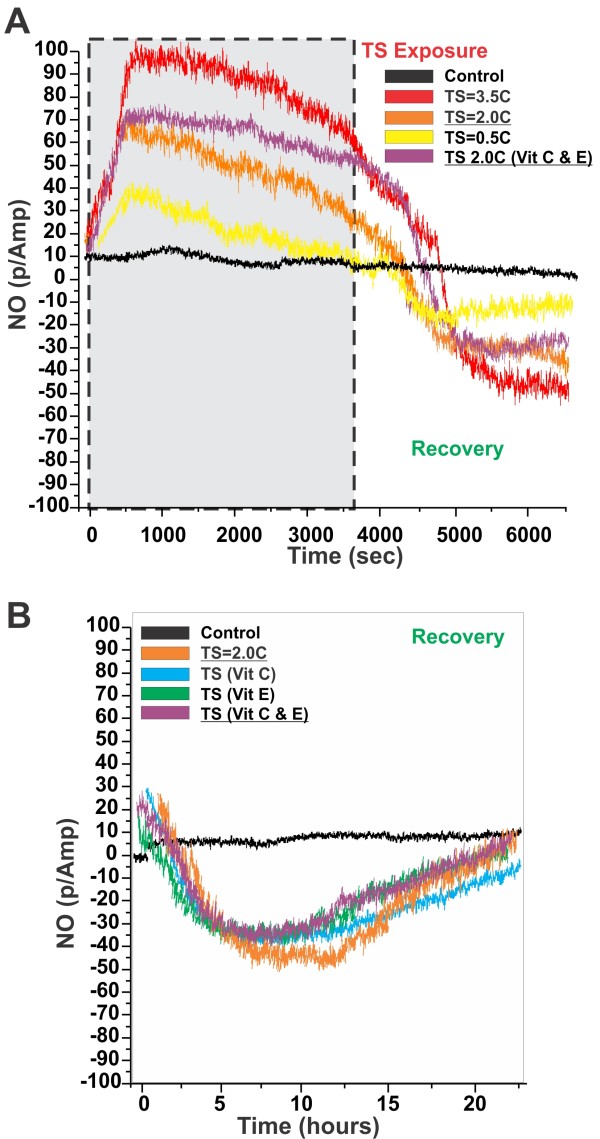
**Amperometric determination of NO during and after TS exposure**. (**Panel A**) Note the transient surge in the medium levels of NO immediately after TS exposure followed by a steady decline that brought the NO to below baseline levels during the recovery time. (**Panel B**) Pre-treatment with vitamins C and E did not prevent the reduction of NO bioavailability nor accelerated the return to a baseline level but reduced the NO decline during the initial phase of TS exposure (**Panel A **-right). Please note that reading conversion of the NO sensors was ≅ 1.5pA/nM ± 3%. The described experiments were performed in quadruplicates (n = 4).

NO release by TS and by the vascular endothelium was assessed in real time by amperometric determination through electrode(s) in contact with the luminal and the abluminal compartments of the DIV-BBB modules. We determined a sudden and transient increase in the medium levels of NO (Figure 6A) in BBB modules exposed to TS versus sham systems, which correlated with the concentration of TS (0.5, 2.0, 3.5 cigarettes/hour for 1 hour yielded 40, 60 and 100 p/Amp NO peaks respectively). NO started declining immediately after reaching the peak level. However, the NO decline was negligible in DIV-BBB modules pre-treated with antioxidant vitamins C and E.

During the recovery time (in the absence of TS) we observed a diametric decrease of NO, which also correlated with the concentration of TS to which the corresponding BBB modules were initially exposed (Figure 6A). NO remained below the baseline level for ≈ 23 hours before returning to normal. Pre-treatment with vitamins C and E did not prevent the reduction of NO bioavailability nor accelerated the return to a baseline level (Figure 6B).

### Tobacco smoke can alter the normal BBB bioenergetic metabolism: role of antioxidant vitamins

**Figure 7:**
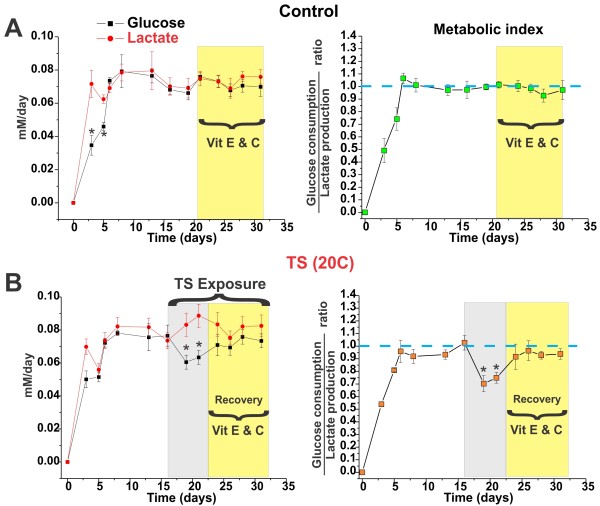
**BBB glucose metabolism is affected by TS exposure**. The ratio between glucose consumption and lactate production is not affected by vitamins supplementation (C+E) (**Panel A**). Note that a glucose consumption/lactate production ratio ≈ 1 indicates that at list 50% of glucose undergoes oxidative metabolism (Krebs cycle). TS caused a decrease in glucose consumption paralleled by an in lactate production (**Panel B**) suggesting that a shift toward a non-oxidative glucose metabolism occurred. This metabolic shift was gradually reversed by antioxidant vitamins supplementation (C+E). The asterisk "*" indicates a statistically significant difference (p < 0.05) versus control. The described experiments were performed in quadruplicates (n = 4).

Under normal physiological conditions BBB endothelial cells are characterized by a predominant oxygen-depended bioenergetic metabolism (aerobic) [66,71]. The increased demand for reducing equivalents (e.g., NADH, FADH_2_, etc.; normally utilized during oxidative respiration) to neutralize the reactive oxidative species from TS can cause metabolic alterations in the BBB endothelium [71]. As shown in Figure 7A (left panel) glucose consumption and lactate production levels were stable during the pre-TS phase treatment with a metabolic index (ratio between lactate production and glucose consumption) ≅ 1 (Figure 7A right panel). Administration of vitamins C and E at physiological concentrations (80 μM and 40 μM respectively) did not alter the metabolic behaviour of the BBB. However, exposure to TS (cycles of chronic exposure: every other hour for 12 hours/day) caused a decrease in glucose consumption (see Figure 7B left panel) and an unparalleled increase in lactate production, demonstrating a shift toward predominantly anaerobic bioenergetic metabolism (metabolic index ≅ 0.7; see Figure 7B right panel). Co-administration of vitamins C and E gradually restored the bioenergetic metabolism of the BBB.

## Discussion

The BBB maintains brain homeostasis and selectively excludes most blood-borne and xenobiotic substances from entering the brain, protecting it from systemic and exogenous influences [72,73]. Deterioration in BBB functions have been clearly shown to play a major role in the pathogenesis and progression of a steadily increasing number of neurological disorders such as multiple sclerosis, Alzheimer's disease, neoplasia, hypertension, dementia, epilepsy, infection, and trauma [74]. While the harmful effects of smoking on public health have been well demonstrated and detailed in many organs, the BBB has received much less attention despite the strong evidence for an association between tobacco smoke and vascular impairment. Recently published data [25] have shown that TS negatively affects vascular endothelial physiology and directly promotes the pro-inflammatory activation of BBB endothelium.

Many of the over 4000 components contained in TS are thought to facilitate the development of a proatherosclerotic environment [25,28,75-77] by triggering a complex pro-inflammatory response. This is mediated by the recruitment of leukocytes through cytokine signalling (e.g., IL-2, IL-4, IL-6, IL-8, INF-γ and GM-CSF), matrix metalloproteinase upregulation (e.g., MMP-1, MMP-8, and MMP-9), and by promoting the adherence of monocytes to the endothelial wall [25,78].

TS's status as a powerful pro-inflammatory agent is suggested by the observation of elevated levels of white blood cells, primarily neutrophils and monocytes, in smokers [79]. Elevated C-reactive protein (CRP) levels caused by TS promotes endothelial dysfunction by lowering the production of NO and diminishing its bioactivity [80], thereby limiting the smoker's ability to vasodilate via NO release.

One well accepted yet poorly understood mechanism by which smoking can directly hamper BBB viability and function relays upon oxidative stress caused by the many highly reactive oxygen species and free radicals of which TS is highly enriched. The gas-phase of tobacco smoke alone is a complex mixture of high concentrations of ROS and free radicals (approximately one quadrillion radicals per puff [81]). This represents an exorbitant load of oxidant to body tissues which can lead to BBB breakdown by oxidative damage, tight junction (TJ) modification, and matrix metalloproteinases (MMP) activation [32]. The results presented herein suggest that antioxidant vitamins may provide an effective shielding against oxidative stress thus, protecting the BBB during TS exposure.

Our data have shown that the administration of physiological concentration of vitamin E and/or C reduced the BBB endothelial pro-inflammatory activation solicited by TS exposure (see Figure 1). While the precise mechanism/s by which TS triggers an immune vascular response at the BBB cannot be determined from these experiments, our data strongly suggest that: 1) the imbalance between tissue/plasma concentrations of antioxidants and reactive oxygen species caused by TS played a major role in the process; 2) this can be partially prevented by administration of antioxidant vitamins.

The BBB endothelium is not the only tissue affected by TS. Our data also showed that TS directly activated monocytes which resulted in the release of IL-1β and TNF-α and MMPs (see Figure 2). We also observed a time dependent increase in the activity of MMP-2 and MMP-9 which initiated as early as 15 minutes of TS exposure and increased substantially after 60 minutes. The increased activity of matrix metalloproteinases reflected the continuous release of these enzymes during TS exposure suggesting an ongoing pro-inflammatory stimulation of the cells. Similar to what observed in BBB ECs cultures, pre-treatment with antioxidant vitamins reduced monocytes pro-inflammatory activation by TS.

In this study we used a final concentration of vitamin C (80 μM) equivalent to that found independently of gender in healthy non-smoker subject (35.3-107.1 μM; 6.2-18.8 mg/L ) according to literature data [82]. Not surprisingly the anti-inflammatory activity of vitamin C was limited to the initial 15 minutes of TS exposure. Vitamin C is an hydrophilic reducing agent making it the first line of defense against ROS and the first to be consumed in the process. Therefore, it very likely that higher concentrations and/or a sustained supplementation of vitamin C are necessary to maintain a protective effect for a longer period of time. This is agreement with the decision of Food and Nutrition Board of the National Academy of Sciences to raise the RDA of vitamin C for smokers to over 200 mg/day (≅ 160 μM/l cconsidering an estimated 70% absorption which decreases as intake increases) versus the recommended 90 mg/day for non-smokers. This help counteracting vitamin C depletion caused by TS-derived ROS over time.

On the other hand, when vitamin C was co-administered with vitamin E (a powerful fat soluble reducing agent and the primary antioxidant present in the lipid-rich cell membranes; see also Figure 3) the protective effect was significantly enhanced both in efficacy and duration. The interaction between vitamins E and C has been recognized and extensively studied showing an ascorbate-mediated regeneration of oxidized membrane alpha-tocopherol back to its reduced form. A process that extends the protective effect of vitamin E against oxidative stress [83].

Whether ROS can directly hamper BBB viability was also clearly demonstrated. Our results showed a time and dose dependent loss of BBB viability when in the absence of physiological antioxidants protection the DIV-BBB modules were exposed to H_2_O_2 _levels comparable to those a chronic smoker is routinely exposed (see Figure 5). Our findings are in agreement with studies by others showing that plasma antioxidant vitamins (especially ascorbic acid) levels in chronic smokers are significant decreased due to accelerated turnover in response to TS-evoked oxidative stress [51]. This is also in agreement with the fact that administration of antioxidant vitamins C and E (particularly when combined) prevented the loss of BBB integrity.

Not surprisingly, direct BBB exposure to TS showed similar results. We observed a similar time and dose dependent pattern of BBB impairment (see Figure 4A), which was paralleled by the release of adenylate kinase (a well-established marker of cell damage; see Figure 4B). This suggests that the increased paracellular permeability is not exclusively dependent (at least in the short term) on the exposure to nicotine [84] contained in TS but also to cell damage caused by ROS. This hypothesis is supported by the fact that pre-treatment with antioxidant vitamins significantly reduced the loss of BBB viability during TS exposure (see Figure 4C). This was accompanied by a similar decreased in the release of AK (see Figure 4D) and that of pro-inflammatory cytokines (see Figure 4E). The latter is also in agreement with previous studies showing that vitamin C plays an important (yet not fully understood) role in the modulation of cytokines release as well as other immune surveillance activities [57].

Another mechanism through which tobacco smoke can impair BBB viability is through the alteration of nitric oxide bioavailability. NO is a molecular mediator involved in a variety of physiologic and pathological processes. Three isoforms of NOS have been described: neuronal NOS (nNOS), endothelial (eNOS) and inducible or immunological NOS (iNOS). Vascular endothelial cells generate small bursts of NO from L-arginine via increase in intracellular Ca^2+ ^through a calcium-dependent constitutive NO synthase (eNOS). In contrast, iNOS induced in pathological/inflammatory state is Ca-independent manner and releases NO continuously in large amounts. Our data showed a sudden burst in NO (see Figure 6A) immediately after the initial exposure to tobacco smoke. Consequent to the sudden raise of NO we observed a steady decline on NO levels which suggests a rapid reaction of NO with other TS-released ROS and its conversion in peroxynitrite (ONOO-) [85]. This hypothesis is in agreement with the fact that NO decline was prevented by pre-administration of antioxidant vitamins, which neutralized the ROS contained in TS. This being the case, it is possible that antioxidant treatments may indeed prolong the initial NO-dependent vasodilatation experience by smokers during TS exposure.

By contrast, during the recovery time (in the absence of TS) NO dropped below the baseline level. The magnitude of this decline appeared to be proportional to the amount of TS to which the corresponding BBB modules were previously exposed. While the precise mechanisms by which TS affected NO bioavailability cannot be entirely determined from these experiments, some inferences can be still derived. Nicotine contained in TS has been shown to inhibit eNOS activity, thus decreasing the constitutive level of NO release by the vascular endothelium [86,87]. As shown in Figure 6B pre-treatment with antioxidant vitamins did not affect the decline of NO during the recovery phase. This supports the hypothesis that perhaps nicotine rather than ROS are responsible for the observed decline of NO during this phase leading to vasoconstriction and aggravating the vascular impairment of the brain [88,89].

Tobacco smoking has also been associated with impairments of glucose metabolism and glycemic regulation [90]. Our data have clearly shown that TS exposure determined a decrease in glucose consumption (Figure 7B left panel), paralleled by an increase in lactate production. This is indicative of a potential change in the BBB bioenergetic behaviour toward a more predominant anaerobic metabolism (see Figure 7B right panel). The mechanisms through which TS affect glucose metabolism may be related to free radicals and other ROS contained in tobacco smoke [11]. In vivo, this effect can be further exacerbated by the carbon monoxide (CO) also contained in tobacco smoke. CO through binding to hemoglobin, myoglobin and mitochondrial cytochrome oxidase [91]can cause a relatively prolonged impairment of glucose oxidative metabolism and a decrease in glucose utilization [92,93]

One way through which BBB endothelial cells can try to counteract the increased levels of ROS is through the generation of reductant equivalents such as glutathione peroxidase (of the principal antioxidant defense enzymes). Generation of glutathione peroxidase requires oxidized glutathione to be reduced by glutathione reductase using NADPH generated in the pentose phosphate pathway (also called the phosphogluconate pathway). This process generates NADPH (oxidative phase) and pentoses (5-carbon sugars; non-oxidative synthesis) [11]. A shift toward the pentose phosphate pathway to compensate for the excess of ROS would explain the reduced glucose consumption we observed during TS exposure.

Furthermore, BBB endothelial cells could also use the Krebs cycle reducing agents (normally utilized to sustain the energy-producing pathway based on oxidative phosphorylation) to counteract TS-derived ROS. This would leave the cellular mitochondria underequipped to sustain the oxidative glucose metabolism and would explain the increased lactate production also observed during TS exposure. This hypothesis is supported by our data which showed that administration of antioxidant vitamins C and E gradually restore the normal bioenergetic metabolism of the BBB (see Figure 7B) [11,94]. However, a more specific and detailed study will be necessary to confirm this hypothesis and unravel the underlining mechanism/s.

## Conclusion

Our data indicate that loss of endothelial viability as well as BBB function and integrity *in vitro *caused by TS exposure can be significantly reduced by pre-treatment with physiologic concentrations of α-tocopherol and ascorbic acid. These antioxidant vitamins may act synergistically in preventing oxidative damage and pro-inflammatory stimulation induced by tobacco smoking exposure, thereby reducing TS toxicity at the BBB level.

### Study limitations

In vitro toxicology studies of tobacco and tobacco smoke have been used widely used to assess the toxicologic impact of tobacco product. However, there are limitations with respect to the generality of the findings inherited in such studies which reflect the inability of any *in vitro *model to fully recapitulate the biological and physiological make up a living organism. In this study we used an artificial vascular system to assess the effect of TS exposure on the BBB and whether administration of vitamin C and/or E can reduce TS toxicity. However, our model cannot reproduce for example, the effect of hepatic clearance and peripheral metabolism on the concentration of TS components to which the BBB and the immune system are exposed. This can lead to a relative over-exposure of the BBB and its constituents (including circulating monocytes) to TS thus, exaggerating its acute harmful effects. *In vitro *studies on TS toxicity (such the one reported in here) more likely reflect the impact of chronic smoking over a long period of time (in general several years) were cumulative harmful effect builds up overtime.

## Methods

### TS Preparation

Concentrated smoke solution was prepared from 2R4F research cigarettes, which are high nicotine, high tar, filter cigarettes (University of Kentucky). The cigarettes were smoked using a Borgwaldt RM2 apparatus according to ISO determination parameters. These require a puff volume for each cigarette of 35 mL with duration of 2s at intervals of 60s, with an airflow surrounding the cigarette of ≅ 200 ± 30 mm/s. This protocol resulted in approximately 8 puffs/cigarette. Stocks of smoke solution were obtained by drowning (using a vacuum pump) the mainstream smoke from 40 cigarettes through 10 ml of sterile phosphate buffered saline (PBS) (abbreviated TS1). Tobacco smoke was then quantified as cigarette/mL (c/mL). The resulting concentration of the stoke solutions was ≅ 4c/mL. The stock solutions were used immediately after preparation. Note that cells were put in contact with the soluble smoke extract in cycles of 120 minutes for a total period of 12 hours/day where each cycle was divided as described: 0-15 minutes (vitamin pre-treatment or nothing); 15-75 minutes (TS exposure or nothing if control), 75-120 exchange with TS-free medium (including controls). Each set of cycles was designed to deliver exposure to TS equivalent to that yielded by ≅ 20 cigarettes per day or ≅ 1.67c/cycle. Media samples (TS and TS-free) were collected throughout the cycles and stored for analysis.

### Hydrogen peroxide preparation

Hydrogen peroxide solution (PERDROGEN^® ^30% H_2_O_2_) was purchased from Sigma-Aldrich (St. Louis, MO 63178; cat# 31642) and diluted to achieve the appropriate final experimental concentrations (55, 220, and 385 μM respectively which are comparable to that yield by 5, 20, and 35 2R24 research cigarettes; 2-4 μM/mg of tar [69,70]). Dulbecco's Phosphate Buffered Saline (Sigma-Aldrich, St. Louis, MO 63178; cat# D4031) containing 36 mg sodium pyruvate, 50 mg streptomycin sulfate, 100 mg kanamycin monosulfate, 1000 mg glucose/L and CaCl_2 _adjusted to a pH ≅ 7.5 was used to make dilutions of hydrogen peroxide. The solutions were prepared fresh before the experiment and maintained at 37°C.

### Ascorbic acid and α-tocopherol preparation

α-tocopherol (vitamin E, cat# **AC42103-1000**) and ascorbic acid (vitamin C, cat# **AC40147-1000**), were purchased from Fischer Scientific Pittsburgh, PA. 43.07 mg of α-tocopherol were dissolved in 10 mL of ethanol yielding a stock solution of 10 mM. The experimental concentration of α-tocopherol (40 μM) was achieved by diluting 4 μL of the stock solution in 1 mL of the specific growth medium. Ascorbic acid (35.22 mg) was instead dissolved in 10 mL of PBS yielding a stock solution of 20 mM. Final experimental concentration of ascorbic acid (80 μM ) was achieved by diluting 4 μL of the stock solution in 1 mL of the specific growth medium. In the described experiments cells were pre-treated with vitamin C and E (at a final concentration of 80 μM and 40 μM respectively) prior exposure to soluble tobacco smoke extract.

### Cell Culture

Normal human brain microvascular endothelial cells (HBMEC, cat# 1000), and human astrocytes (HA, cat# 1800) were purchased from ScienCell Research Laboratories, San Diego, CA 92121. HBMEC were expanded in 75 cm^2 ^flasks pre-coated with fibronectin (3 μg/cm^2^) using Endothelial Basal Medium MCDB 105 (Sigma, Cat# M6395) containing 10% human AB serum (SIGMA, Cat# S-7148), 15 mg/100 ml of endothelial cell growth supplement (ECGS, Cat.# 1052), 800 units/ml of heparin (Sigma, cat# H3393), 100 units/ml penicillin G sodium and 100 mcg/ml streptomycin sulfate. Prior to use HBMEC were characterized by immunocytochemistry using sheep polyclonal antibodies that recognized the human Von Willebrand Factor Antigen VIII (vWF/Factor VIII, US biological, Swampscott, MA, cat# F0016-13A).

HA were grown in Poly-d-Lysine pre-coated flasks (3 μg/cm^2^) with Dulbecco's modified essential media (DMEM-F12) supplemented with 2mM glutamine, 5% fetal bovine serum (FBS), 100 units of sodium penicillin G per ml, and 100 mcg of streptomycin sulfate per ml. To obtain a highly purified astrocytic population Cytosine arabinoside and L-leucine methyl ester (Sigma-Aldrich, MO, USA) were added [95] and HA cultures were agitated overnight at 37°C. We used rabbit polyclonal antibodies specific for the glial cell marker glial fibrillary acidic protein (GFAP, Dako Corporation, Carpentaria, CA, USA) to assess the purity of the HA cultures which resulted > 95%. Both endothelial cells and astrocytes were maintained at 37°C in a humidified atmosphere with 5% CO_2_. Note that cells were not expanded for more than 2 cycles to minimize cell dedifferentiation.

The human monocytic leukemia cell line (THP-1, Cat# TIB-202) was purchased from American Type Culture Collection (ATCC, Manassas, VA). THP-1 cells were grown at 37 °C in 95% air-5%CO_2 _in basal medium (ATCC-formulated RPMI-1640 Medium, Catalog No. 30-2001) plus 0.05mM 2-mercaptoethanol and 10% fetal bovine serum as specified by the vendor.

### DIV-BBB setup

**Figure 8:**
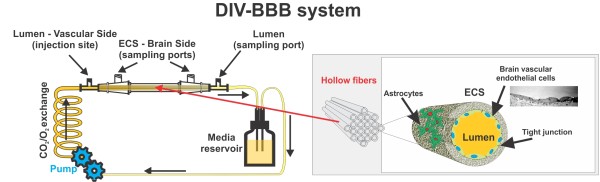
**Schematic of a DIV-BBB module**. A bundle of porous polypropylene hollow fibers is suspended in the DIV-BBB chamber. The artificial capillaries are in continuity with a medium source through a flow path consisting of gas-permeable silicon tubing. Ports positioned on either side of the module allow access to the luminal and abluminal compartments. Note how this artificial capillary system closely mimics the physiology of the BBB *in situ*.

HBMEC and HA were cultured in a dynamic model of the blood-brain barrier (DIV-BBB) in polypropylene or on hollow fiber capillaries, as previously described (see Figure 8). Briefly, the luminal surface of the hollow fibers was pre-coated with 3 μg/cm^2 ^fibronectin to allow HBMEC adhesion. The abluminal surface of the fibers was pre-coated with 3 μg/cm^2 ^of poly-D-lysine to promote HA adhesion. HBMEC were first introduced into the luminal compartment and allowed to adhere for 3 hrs without pulsatile flow. To maximize HBMEC adhesion, the flow of medium was initiated in the abluminal compartment for 24 hrs, then switched to pass directly over the HBMEC in the luminal compartment at a low level shear stress (2 dyne/cm^2^) for another 24 hours. The shear stress was then raised to a constant value of 4 dyne/cm^2^. HA were seeded on the abluminal surface of the fibers three days after HBMEC were loaded. Typically, two weeks of co-culture are required to establish a fully functional BBB with a TEER greater than 800 Ω cm^2 ^above the baseline (measured as TEER in a cell-free module).

### TEER measurement

TEER measurement provides a rapid, simple evaluation of the integrity of the DIV-BBB. Our TEER measurement device was purchased from Flocel (Flocel Inc., Cleveland, OH 44103). This system utilizes electronic multiplexing to test multiple DIV-BBB modules in rapid succession and deliver data to a computer. Details on the core system are available on the website of the maker of the device (http://www.Flocel.com). We have previously verified that this method documents a strong inverse relationship between TEER and permeability across the EC monolayer in the DIV-BBB modules [66-68].

### Measurement of nitric oxide (NO)

Changes in nitric oxide (NO) were measured by amperometric determination using an optically isolated multi-channel free radical analyser (World Precision Instruments, Sarasota, FL). The NO measuring probe is inserted into the lumen downstream from the hollow fiber apparatus and another probe in the ECS compartment. NO is detected as previously described [96,97]. The on-line changes in NO were continuously measured using a PC-driven system. Based on internal calibration reading conversion of the NO sensors was ≅ 1.5pA/nM of NO. Max sensitivity ≅ 50 fA according to the manufacturer.

### Measurement of cytokines and MMPs

Samples of medium from the luminal compartment were centrifuged at 5,000 g for 5 min and stored at -20 °C until analysis. Levels of IL-6, IL-1β, and TNF-α were measured by ELISA (cat# IB39673 (IL-6); # IB39654 (IL-1β; # IB39699 (TNF-α); IBL America, Minneapolis, MN) according to the manufacturer's protocols. MMP-2 and MMP-9 were also quantified by ELISA (cat# QIA63-1EA (MMP-2) and QIA56-1EA (MMP-9); Calbiochem, San Diego, CA). Final calculation of cytokine levels (pg/mL) took into consideration time and volume of the luminal compartment according to the formula: (V_t_xC_c_)-(V_n_xC_n_)+(V_total_xC_p_)/T_c_-T_p_, where V represents added volume of media (mL); C refers to the concentration of a cytokine/MMP level (pg/mL); T is time of sampling (in fraction of days: _c _and _p _indicate the current and previous samples, respectively; and _n _represents cytokine values in the fresh medium added after each sampling.

### Flow cytometry (FACS) and data analysis

TS-exposed and control endothelial cells were rinsed with PBS. Cells were then gently mixed with chelation buffer (1mM EDTA prepared in Ca^2+^/Mg^2+ ^free PBS) using a pipette tip washed in FACS buffer. (PBS+0.5% BSA+0.02% NaN_3_) and resuspended to approx. 1 million cells/100 μl. Cells were incubated with fluorescein di-isothiocyanate conjugated anti-P-selectin, phycoerythrin-conjugated anti-E-selectin, or alkaline phosphatases conjugated anti-VCAM-1 (respectively, cat.#555524; # 551144; #551146; BD Biosciences, San Jose, CA) for 20 minutes in the dark. Unbound antibody was removed by several washes with FACS buffer. Cells were then resuspended in 200 μl of FACS buffer/sample for analysis and data were calculated by Flow Jo 6.1.1 (Tree Star) for Mac OS X. THP-1 cell suspensions were similarly tested for CD45/CD14 (cat. #340040; BD Biosciences)

### Matrix metalloproteinase activity

Aliquots of medium from the luminal compartment were centrifuged at 14,000 g for 15 min at 4 °C and any cell pellet discarded. Total protein content of the medium was determined by Bradford assay. Gelatin zymography of electrophoresed medium samples was carried out on 7.5% polyacrylamide gels copolymerized with 2 g/L 90 Bloom Type A gelatin from porcine skin (Sigma). After electrophoresis, gels were washed in Triton X-100 (2.5 mL/L) and incubated for 24 h (37 °C) in enzyme buffer (50 mM Tris-HCl, pH 7.5; 5 mM CaCl2; 100mM NaCl; 1mM ZnCl2; 0.2 g/L Brij^®^-35; 2.5 mL Triton X-100; and 0.02 g/Lf NaN3). Gels were stained with 0.5% Coomassie Blue R-250. Lysis bands were measured densitometrically with an image analyzer.

### Statistical analysis

For parametric variables (e.g., TEER levels, glucose consumption, lactate production, cytokines levels), differences between populations were analysed by ANOVA. p values < 0.05 were considered statistically significant. Bonferroni analysis was used to account for comparisons of multiple parameters among groups. For non-parametric indices (e.g. densitometries for zymogram), we used the Kruskal-Wallis test followed by Mann-Whitney U-test. We used four cartridges per data point. Based upon previous experiments, this number of cartridges provided sufficient power to demonstrate statistical significance.

## Disclosure/Conflict Of Interest

Dr. Cucullo has reported the following financial relationships with the companies listed below.

**Equity**: Dr. Cucullo owns stocks in Flocel Inc.

## Authors' contributions

MH established all the cell cultures, the DIV-BBB modules and the static co-culture systems. He also performed all the experiments described in this manuscript including the immunoassays and zymographies. PM provided his expertise in data analysis and collaborated with LC in the draft of the manuscript. WT participated in the data analysis and provided substantial support in the editing of the manuscript. LC conceived and supervised the study, elaborated its design and the experimental procedures and drafted the manuscript. All authors have read and approved the final manuscript.
